# Body Composition and Its Correlates in Children and Adolescents Living in Germany: A Cross‐Sectional Study

**DOI:** 10.1002/ejsc.70066

**Published:** 2025-10-06

**Authors:** Raphael Schilling, Steffen C. E. Schmidt, Elena Schlag, Claudia Niessner, Alexander Woll, Janis Fiedler

**Affiliations:** ^1^ Institute of Sports and Sports Science Karlsruhe Institute of Technology Karlsruhe Germany

**Keywords:** fitness, health, physiology, youth

## Abstract

Body composition is an important health parameter during childhood and adolescence. In this study, we investigate the associations between body composition and age, physical activity, side jump, standing long jump, physical working capacity at 170 beats per minute pulse, screen time, and socioeconomic status in a nationwide German sample. A total of 2.869 children and adolescents (1.456 girls) aged 6–17 years from the Motorik‐Modul study (2014–2017) were stratified by sex and three age groups (6–10, 11–13, 14–17 years). Physical activity was quantified by accelerometers, while physical fitness parameters were measured as part of the Motorik‐Modul field‐based fitness test battery. Body composition analysis (BIA 2000‐S; Data Input, Frankfurt, Germany) included phase angle as well as height‐adjusted indices for fat mass and fat‐free mass. Potential correlates of body composition were examined by testing 18 preregistered hypotheses via multiple regression analyses. We found a general increase in fat mass index, fat‐free mass index, and phase angle during childhood with sex‐dependent changes in these trajectories occurring between the ages of 10 and 14 and persisting up to age 17. Besides age, the most important correlates were standing long jump and physical working capacity. Higher screen time and lower socioeconomic status accompany higher fat mass index but do not predict a lower fat‐free mass index. Physical activity correlates negatively with fat mass index only among 6 to 13‐year‐olds. These findings emphasize the complex interplay between body composition, physical fitness parameters, lifestyle factors, and socioeconomic background in childhood and adolescence.

## Highlights

- Body composition indices (fat mass index, fat‐free mass index, phase angle) in German participants increase during childhood. Boys show accelerated fat‐free mass index growth starting around 11 years and phase angle growth starting around 14 years, while girls exhibit a more linear increase across all indices.
- In addition to age, standing long jump and physical working capacity are the strongest predictors of body composition across all age groups.
- Higher physical activity is associated with lower fat mass index specifically in younger children (6–13 years).
- Implementing bioelectrical impedance analysis‐based growth charts for body composition may enhance the understanding of the relationships between growth, health, aging, and disease, supporting both scientific research and clinical practice.

## 1 Introduction

Body composition is an important health parameter in childhood and adolescence (Androutsos and Zampelas 2022), yet it is infrequently assessed and, for example, not included in mandatory medical check‐ups in Germany (Federal Joint Committee 2024). Unhealthy developments, such as excessive gains in fat mass or reduction in fat‐free mass, can reduce quality of life (Meixner et al. 2020) and may increase the risk of developing comorbidities (Lister et al. 2023), with potentially long‐term negative effects on health in adulthood (Simmonds et al. 2016). Despite preventive action plans (World Health Organization 2023), the number of children and adolescents with unhealthy body composition remains high worldwide, with significant increases during the COVID‐19 pandemic in Germany (Vogel et al. 2022), and potentially globally (Stavridou et al. 2021).

Bioelectrical impedance analysis (BIA) is a non‐invasive method to depict the human body using up to a four‐compartment model, instead of the one‐compartment model the body mass index offers. Here, the specific assessment of fat mass (Roubenoff et al. 1995), extracellular mass, and intracellular mass, as well as an assessment of the quality of body cell mass (phase angle (PhA) or ratio between extra‐ and intracellular mass) and total body water allows a more detailed view of the condition of the body (Schmidt et al. 2019). Differentiating between potentially unhealthy fat mass and fitness‐related fat‐free mass is fundamental to our study, as it enables a two‐dimensional investigation of the associations between body composition and key health‐related factors. In the following, we present data using a BIA‐derived two‐compartment model. As advised by other authors (Bosy‐Westphal and Müller 2015; Kyle et al. 2003) we use fat and fat‐free mass indices by dividing fat mass and fat‐free mass by the square of the body height, to eliminate correlations with height and increase the accuracy of age group dependent interpretations. We recently published reference data for fat mass index (FMI) and fat‐free mass index (FFMI) for children and adolescents (Schmidt et al. 2019) and the longitudinal Motorik‐Modul (MoMo) study continues to produce representative data for Germany. In addition to FMI and FFMI, PhA is another meaningful health parameter in children and adolescents (Langer et al. 2020). It provides insights into the body's capacitive impedance, a metric that is fundamentally associated with the mass and structural integrity of cells, thus acting as a vital biomarker in clinical settings (Stobäus et al. 2010).

Findings from previous studies using body mass index suggest that several factors (e.g., physical activity (PA), physical fitness (PF), screen time (ST), socioeconomic status (SES)) are directly and indirectly linked to body composition during childhood and adolescents and might play a role in developing or preventing an unhealthy body composition (Elmesmari et al. 2018; Sares‐Jäske et al. 2022; Tripathi and Mishra 2020).

These results are confirmed by several studies employing BIA to assess body composition. In children and adolescents, higher levels of PA (Dowda et al. 2024; Ito et al. 2021), PF (Gualdi‐Russo et al. 2020; Joensuu et al. 2020), and higher SES (Van den Berg et al. 2013) are associated with lower levels of body fat and higher levels of fat‐free mass. Increased ST is linked to higher levels of body fat (Chaput et al. 2014). Regarding PhA in children and adolescents, studies showed that higher PhA values are associated with higher levels of PF (Langer et al. 2020; Martins et al. 2020). In addition, associations were also found with age, sex, maturity, and height (Ballarin et al. 2022; De Moraes et al. 2022). Research about PhA has been expanding as well (De Moraes et al. 2022), but there remains insufficient evidence regarding its true potential and relevance during childhood and adolescence (Orsso et al. 2022). There is also a lack of large, representative studies that assess and analyze a multitude of different health parameters such as PF, PA, or ST and their intercorrelation with body composition at the same time and sample.

The comprehensive range of constructs captured in the MoMo study, coupled with the substantial volume of data available for analysis, provides a unique opportunity for scientific inquiry. To take this into account, we have opted to employ a confirmatory approach to investigate the associations between body composition and health‐related correlates among children and adolescents. The aim of the study was to provide data on body composition derived from BIA of a nationwide German sample and to analyze its associations with three selected PF parameters, as well as other health‐related constructs such as age, PA, ST, and SES using regression analyses. We preregistered our study (Schilling et al. 2023) and formulated 18 directed hypotheses derived from the current state of research. We hypothesized that higher PA (Gualdi‐Russo et al. 2020; Mateo‐Orcajada et al. 2022), higher performance in the specific PF tests (Androutsos and Zampelas 2022; Ballarin et al. 2022; Ortega et al. 2008), and higher SES (Lizana et al. 2018), as well as lower ST (Stiglic and Viner 2019), would be associated with more favorable body composition outcomes (i.e., lower FMI, higher FFMI, and higher PhA). These hypotheses cover the potential relationship between the variables of interest and will be tested separately for boys and girls among three age groups (6–10 years, 11–13 years, 14–17 years). Notably, our hypotheses are tested within separate models rather than a single comprehensive model incorporating all predictors. This approach aligns with prior research, which predominantly examined bivariate relationships rather than complex multivariate interactions.

## 2 Materials and Methods

Our study followed the Declaration of Helsinki. Ethics approval was obtained from the ethics committees. The Federal Commissioner for Data Protection approved the study. This paper was preregistered at the open science framework (Schilling et al. 2023).

### 2.1 Study Design

Data were collected within the MoMo study (Woll et al. 2021) using a nationwide, stratified, multi‐stage sample (Kamtsiuris et al. 2007). A systematic sample of 167 primary units was drawn from German communities, stratified by urbanization and geography using the BIK classification (Kurth et al. 2008). Communities were selected proportional to their population of residents under 18 years. An age‐stratified sample was then drawn from official registers, with the aim of achieving 100 participants per sex and per year of age in the final sample. Parents and children attended examination rooms near their homes, with parental consent required for minors. Participation was voluntary, and participants received a 20 € gift. Data protection and study details were provided, with written consent obtained.

### 2.2 Sample Description

For the current analysis, data from participants aged 6–17 years from MoMo Wave 2 (2014–2017) were used. To investigate associations with body composition, only participants with complete and valid data on age, sex, FMI, FFMI, and PhA were included in the analyses (*n* = 2.869). BIA data were considered valid if PhA > 4 and PhA < 9.5, according to international standards (Mattiello et al. 2020) and previous studies (Gätjens et al. 2021). In total, 29 participants with implausible values were excluded. The sample was divided into three age groups (6–10 years: primary school/children; 11–13 years: lower secondary school/early adolescents; 14–17 years: upper secondary school/adolescents) based on completed years of life and stratified by sex (boys: 6–10 years [*n* = 416], 11–13 years [*n* = 432], and 14–17 years [*n* = 565]; girls: 6–10 years [*n* = 403], 11–13 years [*n* = 458], and 14–17 years [*n* = 595]).

### 2.3 Measures

#### 2.3.1 Physical Activity

PA was assessed using an accelerometer (Actigraph GT3Xþ/wGT3X‐BT). Detailed information about the procedure can be found elsewhere (Burchartz et al. 2020). Accelerometers were worn above the hip on the right side of the body over 7 consecutive days. Data were recorded with a 30‐Hz sampling frequency and 1‐s epoch length using the normal Acti‐Graph GT3X filter. Non‐wear time was defined as consecutive zero/nonzero counts over 90 min with an allowance of a 2‐min period of non‐zero counts with an up/downstream 30‐min period of zero counts. Recording of at least 8 h per day was considered valid, and a week had to consist of at least four valid days including at least one valid weekend day to be considered valid (Sherar et al. 2011). These inclusion criteria are consistent with the requirements for inclusion in the International Children's Accelerometry Database (ICAD) (Sherar et al. 2011). To define moderate to vigorous PA (MVPA), specific cut‐off points for the 1‐s epochs of vertical‐axis intensity were used (Burchartz et al. 2023). For children from 6 to 9 years, the cut‐offs from Evenson et al. (2008) were used: Sedentary ≤ 2; light PA > 2 to ≤ 38; MVPA > 38. For children aged 10 and older, the cut‐offs from Romanzini et al. (2014) were used: Sedentary ≤ 3; light PA > 3 to ≤ 40; MVPA > 40.

#### 2.3.2 Physical Fitness

The MoMo Motor Performance Test (Worth et al. 2015) was designed to assess PF in children and adolescents under one‐on‐one supervision. It demonstrated high reliability (*r* = 0.97, *p* > 0.001) with no significant differences in mean values. Objectivity was also strong (*r* = 0.98–0.99), with less than a 1% difference across test items (Oberger et al. 2006). To examine the associations between parameters of PF and body composition, three specific tasks from the MoMo Motor Performance Test were used. The first task involved a submaximal exercise test on a bicycle ergometer to assess cardiovascular endurance. This test measured aerobic capacity using a sequential step test design, starting at 25 W and a cadence of 70 revolutions per minute. Each load level is maintained for 2 minutes, followed by an increase of 25 W (Worth et al. 2015). The achieved power output at a heart rate of 170 beats per minute (Physical Working Capacity (PWC170)) was recorded. The test is terminated under three conditions: (1) Pulse exceeds 190 for participants aged 10 years or younger, or 180 for those aged 11 years or older; (2) cadence drops below 50 revolutions per minute for more than 20 s; or (3) participants choose to stop due to subjective exhaustion. The second task, a standing long jump, measured explosive muscular strength of the lower extremities. Participants perform a countermovement jump, and the distance from the starting line to the first ground contact is recorded. Performance is based on the best of two jumps without falling backward. The third task, a side jump, assesses whole‐body coordination under time pressure. Participants jump between two adjacent 50 cm squares for 15 s, avoiding the boundaries. After a 1‐min pause, a second trial is conducted, with the best score from the two trials recorded based on the number of correct jumps.

#### 2.3.3 Screen Time

ST was measured through self‐reported ST behaviors, a method comparable to direct measures of sedentary behavior in relation to health outcomes (Schmidt et al. 2020a). Participants reported the time spent watching television, gaming, and using the internet recreationally on a seven‐point scale, with responses ranging from “almost never” (1), “15 min per day” (2), “30 min per day” (3), “1 h per day” (4), “2 h per day” (5), “3 h per day” (6), to “4 h per day” (7) for both weekdays and weekend days. An index reflecting the 5:2 ratio of weekdays to weekend days was then calculated for each activity. Total ST was calculated by summing minutes across activities if data for all activities were provided (Schmidt et al. 2020a).

#### 2.3.4 Socioeconomic Status

Individual‐level SES was defined according to the educational and professional status of the parents as well as the total household income per household member (Winkler and Stolzenberg 2009). Education and professional status were asked separately for both parents with the higher score being used (Lampert et al. 2014). Adolescents with parents living in separate households were assigned the SES of the parent they lived with. The three aspects: income, educational status, and professional status were scored on a scale from 1 to 7 according to national reference data, and a sum score was created (range: 3–21) (Lampert et al. 2014, 2018).

#### 2.3.5 Body Composition

BIA was conducted by trained investigators following ESPEN guidelines (Kyle et al. 2004). Participants were nonpregnant, healthy children and adolescents without any conditions affecting fluid balance (e.g., renal, endocrine, or myocardial diseases). Body weight was measured to the nearest 0.1 kg with a calibrated scale (SECA, Birmingham, UK) and standing height to the nearest 0.5 cm using a stadiometer (SECA) with the subject wearing light clothes and no shoes. Body mass index was calculated using the formula body mass index = weight [kg]/(height [m])^2^ and classified according to Kromeyer's percentile‐based classification for children and adolescents (Kromeyer‐Hauschild et al. 2001). Data collection occurred between 8 a.m. and 6 p.m. prior to motor performance tests. Though some BIA guidelines recommend fasting for 2–8 h (Kyle et al. 2004), recent studies show fasting is unnecessary for valid results (Artero et al. 2023; Korzilius et al. 2023). Therefore, fasting was not required. Participants on medication were excluded. Tetrapolar BIA measurements of resistance (R) and reactance (Xc) were taken at 50 kHz between the right wrist and ankle using a BIA 2000‐S analyzer (Data Input, Frankfurt, Germany). Measurements were conducted with participants lying supine on a nonconductive surface, ensuring no contact with metal objects. After resting in a supine position for at least three minutes, R and Xc were measured in 15‐s intervals until stable readings were achieved. The BIA device has a technical error of < 0.5% (Dittmar 2003) and high retest reliability (*r* > 0.82, ICC > 0.96) (Talma et al. 2013). With *r* = 0.96 the validity of estimating body cell mass is high (reference: DXA; (Sergi et al. 2015)). The validity of estimating fat mass lies between *r* = 0.88 (reference: air displacement plethysmography (Von Hurst et al. 2016)); and *r* = 0.92 (reference: DXA; (Von Hurst et al. 2016)). Fat‐free mass, body cell mass, and fat mass were calculated using NutriPlus software (Dörhöfer and Pirlich 2007) and normalized by height^2^ (Heymsfield et al. 2007). In the following FFMI (fat‐free mass/height^2^), FMI (fat mass/height^2^), and PhA as of 57.297*arctan (R/Xc) are reported.

### 2.4 Statistical Analysis

The IBM SPSS Statistics package (Version 28.0) was used for the analysis. Regression models were performed to examine the cross‐sectional associations of age, PA, PF parameters, ST, and SES with FMI, FFMI, and PhA. We examined both linear and quadratic effects of age. The inclusion of age^2^ in the polynomial regression models did not result in a significant increase in the adjusted coefficient of determination (*R* ^2^). The final models consisted of age (Model 0), MVPA (Model 1), side jump, standing long jump, PWC170 (Model 2), ST (Model 3), and SES (Model 4) with FMI, FFMI, and PhA as the outcomes. Age was also included as an additional predictor in Model 1 (Model 1.2), Model 2 (Model 2.1), Model 3 (Model 3.1), and Model 4 (Model 4.1). Lastly, in Model 5, all predictors were considered in one model. Each model was constructed independently, meaning that the inclusion of predictors in one model did not imply that they were carried forward to subsequent models. Rather, each model represents a distinct analytical approach that incorporates specific sets of predictors. The analyses were carried out separately for each sex and age group.

## 3 Results

### 3.1 Descriptive Statistics

A total of 2.869 children and adolescents (1.456 girls, 1.413 boys) participated in our study. The main characteristics of the sample are presented in Table 1.

**TABLE 1:** Descriptive characteristics of the participants divided by sex and age.

	Boys				Girls			
*N* = 2.869	6–10	11–13	14–17	6–17	6–10	11–13	14–17	6–17
	yrs.	yrs.	yrs.	yrs.	yrs.	yrs.	yrs.	yrs.
Case (*n*)	416	432	565	1.413	403	458	595	1.456
Age (yrs.)	7.97	12.00	15.40	12.17	8.02	12.05	15.49	12.34
	±1.41	±0.81	±1.10	±3.26	±1.43	±0.81	±1.07	±3.24
Height (cm)	133.72	157.66	176.63	158.20	133.50	157.22	165.60	154.08
	±10.08	±9.80	±8.41	±20.00	±10.99	±8.48	±6.58	±15.76
Weight (kg)	30.33	48.66	68.27	51.11	30.86	49.11	59.75	48.41
	±8.07	±12.15	±14.02	±19.74	±8.91	±12.17	±11.01	±16.00
BMI (*n*)	416	432	565	1.413	403	458	595	1.456
UW (s) (*n*)	10 (2.4%)	14 (3.2%)	11 (1.9%)	35 (2.5%)	9 (2.2%)	12 (2.6%)	17 (2.9%)	38 (2.6%)
UW (*n*)	20 (4.8%)	18 (4.2%)	21 (3.7%)	59 (4.2%)	24 (6.0%)	33 (7.2%)	18 (3.0%)	75 (5.2%)
NW (*n*)	339 (81.5%)	333 (77.1%)	448 (79.3%)	1120 (79.2%)	309 (76.7%)	341 (74.5%)	468 (78.7%)	1118 (76.7%)
OW (*n*)	30 (7.2%)	46 (10.6%)	36 (6.4%)	112 (7.9%)	33 (8.2%)	43 (9.4%)	52 (8.7%)	128 (8.8%)
OB (*n*)	17 (4.1%)	21 (4.9%)	49 (8.7%)	87 (6.2%)	28 (6.9%)	29 (6.3%)	40 (6.7%)	97 (6.7%)
BIA (*N*)	416	432	565	1.413	403	458	595	1.456
FMI (kg/m^2^)	2.88	4.19	4.29	3.84	3.53	4.84	6.20	5.03
	±1.62	±2.27	±2.41	±2.25	±1.86	±2.30	±2.26	±2.43
FFMI (kg/m^2^)	13.86	15.19	17.52	15.73	13.51	14.86	15.56	14.77
	±1.38	±1.92	±2.10	±2.42	±1.44	±1.93	±1.76	±1.92
PhA (°)	5.79	5.98	6.68	6.21	5.79	5.97	6.27	6.04
	±0.54	±0.57	±0.71	±0.74	±0.53	±0.63	±0.68	±0.66
PA (*n*)	313	300	354	967	316	344	409	1.069
MVPA (min/day)	74.15	52.91	45.79	57.18	58.07	43.20	37.50	45.41
	±25.08	±22.24	±22.38	±26.19	±19.67	±18.50	±17.09	±20.21
PF (*n*)	414	429	561	1.404	399	457	586	1.442
SJ (counts)	24.63	35.05	39.41	33.73	25.70	34.28	37.02	33.02
	±7.37	±6.47	±6.40	±9.11	±7.47	±6.94	±6.00	±8.19
SLJ (cm)	128.46	158.67	192.57	163.31	123.65	149.31	153.80	144.03
	±21.71	±24.05	±27.66	±36.44	±23.29	±23.12	±24.64	±26.98
PWC170 (W)	71.03	106.27	150.23	116.76	60.86	89.74	105.91	90.10
	±20.32	±28.02	±41.30	±46.32	±14.40	±23.10	±29.26	±29.99
ST (*n*)	376	407	545	1.328	361	436	576	1.373
ST [min/day]	104.04	233.15	324.22	233.97	85.35	211.72	289.69	211.20
	±83.65	±166.47	±185.69	±180.89	±73.27	±159.11	±172.00	±169.08
SES (*n*)	415	430	560	1.405	400	454	589	1.443
SES (score)	14.42	13.97	13.72	14.00	14.51	14.04	13.38	13.90
	±3.96	±3.93	±3.83	±3.90	±3.83	±3.69	±3.75	±3.78

Abbreviations: ±, standard deviation; °, degree; BIA, bioelectrical impedance analysis; BMI, body mass index; cm, centimeter; FFMI, fat‐free mass index; FMI, fat mass index; kg, kilogram; m, meter; min, minutes; MVPA, moderate to vigorous physical activity; n, sample size; NW, normal weight; OB, obesity; OW, overweight; PA, physical activity; PF, physical fitness; PhA, phase angle; PWC170, physical working capacity 170; SES, socioeconomic status; SJ, side jump; SLJ, standing long jump; ST, screen time; UW (s), severe underweight; UW, underweight; W, watt; yrs., years.

### 3.2 Associations With Age

**FIGURE 1:**
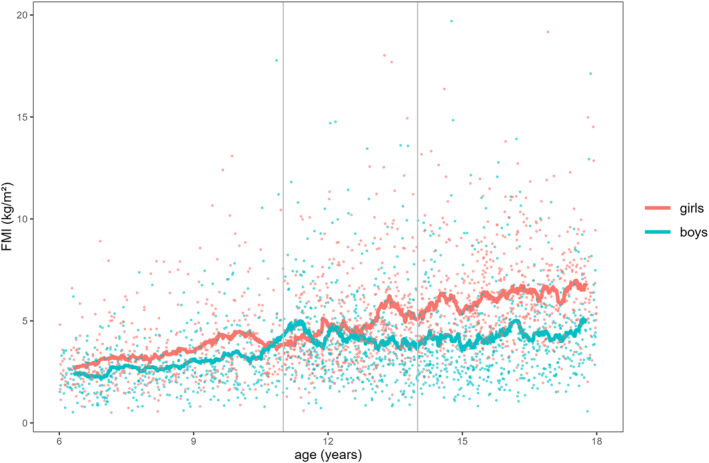
Aggregated fat mass index (FMI) data for both sexes during childhood and adolescence.

**FIGURE 2:**
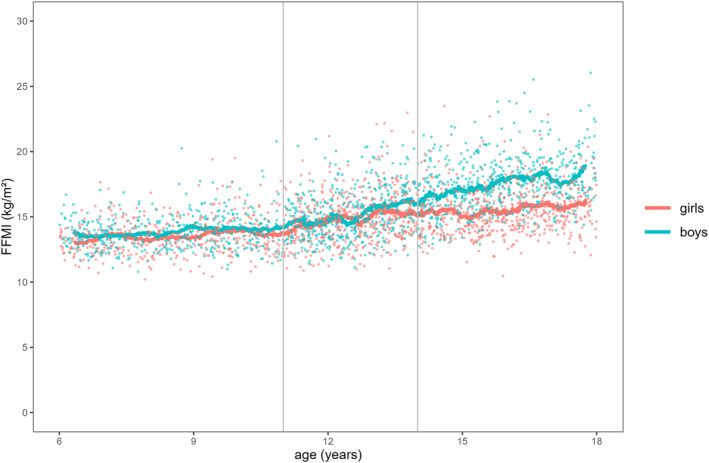
Aggregated fat‐free mass index (FFMI) data for both sexes during childhood and adolescence.

**FIGURE 3:**
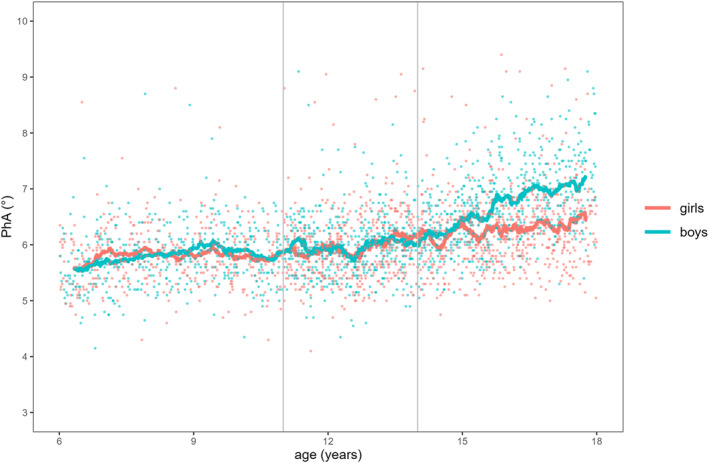
Aggregated phase angle (PhA) data for both sexes during childhood and adolescence.

Figures 1, 2, 3 illustrate the descriptive associations between age and FMI, FFMI, and PhA for both sexes. The smoothing curve represents the 50 data‐points simple moving average (SMA_50_). For FMI (Figure 1), the scatter plot shows higher values with increasing age in both sexes. From 6 to 10 years, girls had a higher FMI than boys of about 0.7 kg/m^2^. Between the ages of 11 and 13, the FMI of boys decreased and then stayed almost at the same level until the age of 17. In contrast, there was a steady increase in the FMI of girls across all age groups. FFMI (Figure 2) showed almost no change for boys and girls between the ages of 6 and 10 until an increase occurred between the ages of 11 and 13 for both sexes. Between the ages of 14 and 17, we found only a slight increase in FFMI in girls but a meaningful increase among boys. For PhA (Figure 3), we found a slight numerical increase for both sexes among the first two age groups. A significant increase was then found among boys between the ages of 14 and 17.

### 3.3 Health‐Related Correlates of Body Composition

Regression models for FMI, FFMI, and PhA are presented in Tables 2 and 3. The models were calculated stratified by sex (boys/girls) and age groups (6–10 years, 11–13 years, 14–17 years).

**TABLE 2:** Unstandardized regression coefficients (*β*) of age, PA, PF, ST, and SES in relation to FMI, FFMI, and PhA among boys.

Boys	6–10 yrs.			11–13 yrs.			14–17 yrs.		
	FMI	FFMI	PhA	FMI	FFMI	PhA	FMI	FFMI	PhA
Model 0									
Age	0.257^a^	0.127^a^	0.172^a^	−0.107^a^	0.302^a^	0.058	0.095^a^	0.303^a^	0.487^a^
*R* ^2^	6.4%	1.4%	2.6%	0.9%	8.9%	0.1%	0.7%	9.0%	23.6%
Model 0.1									
Age	−0.438	0.122	2.226^a^	−0.747	−2.272	−2.557	−1.102	1.695	2.345^a^
Age^2^	0.697	0.005	−2.059^a^	0.639	2.575	2.616	1.198	−1.393	−1.859
*R* ^2^	6.4%	1.1%	4.8%	0.7%	9.3%	0.5%	0.7%	9.0%	23.8%
Model 1									
MVPA	−0.176^a^	0.003	0.129^a^	−0.152^a^	−0.190^a^	0.211^a^	0.000	0.129^a^	0.144^a^
*R* ^2^	2.8%	0.0%	1.3%	2.0%	3.3%	4.2%	0.0%	1.4%	1.8%
Model 1.1									
Age	0.239^a^	0.111	0.186^a^	−0.191^a^	0.256^a^	0.094	−0.074	0.268^a^	0.475^a^
MVPA	−0.127^a^	0.026	0.168^a^	−0.204^a^	−0.121^a^	0.237^a^	−0.008	0.100	0.093^a^
*R* ^2^	8.0%	0.5%	4.4%	5.0%	9.1%	4.6%	0.0%	8.2%	24.0%
Model 2									
SJ	0.108	−0.031	0.096	−0.027	−0.077	0.079	0.012	−0.043	0.096
SLJ	−0.471^a^	−0.175^a^	0.199^a^	−0.568^a^	−0.053	0.155^a^	−0.516^a^	−0.135^a^	0.249^a^
PWC 170	0.336^a^	0.454^a^	0.160^a^	0.153^a^	0.469^a^	0.167^a^	0.191^a^	0.456^a^	0.232^a^
*R* ^2^	18.7%	17.1%	11.9%	28.6%	19.3%	8.5%	22.7%	17.6%	19.5%
Model 2.1									
Age	0.357^a^	−0.025	−0.144^a^	0.087	0.252^a^	−0.070	0.232^a^	0.225^a^	0.345^a^
SJ	−0.047	−0.020	0.159^a^	−0.031	−0.091	0.083	−0.005	−0.060	0.071
SLJ	−0.491^a^	−0.173^a^	0.207^a^	−0.592^a^	−0.124^a^	0.175^a^	−0.560^a^	−0.178^a^	0.184^a^
PWC 170	0.218^a^	0.463^a^	0.208^a^	0.139^a^	0.428^a^	0.178^a^	0.129^a^	0.396^a^	0.140^a^
*R* ^2^	25.8%	16.8%	12.8%	29.0%	24.5%	8.7%	27.1%	21.7%	29.3%
Model 3									
ST	0.256^a^	0.147^a^	0.079	0.034	0.084	0.067	0.145^a^	0.082	0.009
*R* ^2^	6.3%	1.9%	0.4%	0.0%	0.5%	0.2%	1.9%	0.5%	0.0%
Model 3.1									
Age	0.182^a^	0.086	0.150^a^	−0.117^a^	0.318^a^	0.059	0.079	0.301^a^	0.490^a^
ST	0.200^a^	0.121^a^	0.033	0.063	0.006	0.053	0.140^a^	0.063	−0.022
*R* ^2^	9.1%	2.3%	2.1%	0.9%	9.8%	0.3%	2.4%	9.4%	23.6%
Model 4									
SES	−0.126^a^	−0.026	0.000	−0.190^a^	−0.053	0.016	−0.149^a^	−0.143^a^	−0.026
*R* ^2^	1.3%	0.0%	0.0%	3.4%	0.0%	0.0%	2.0%	1.9%	0.0%
Model 4.1									
Age	0.261^a^	0.129^a^	0.178^a^	−0.092	0.308^a^	0.061	0.097^a^	0.306^a^	0.489^a^
SES	−0.134^a^	−0.030	−0.006	−0.182^a^	−0.078	0.011	−0.146^a^	−0.133^a^	−0.011
*R* ^2^	8.0%	1.2%	2.7%	4.0%	9.3%	0.0%	2.8%	11.1%	23.7%
Model 5									
Age	0.250^a^	−0.112	−0.186^a^	−0.019	0.141^a^	−0.122	0.273^a^	0.187^a^	0.349^a^
MVPA	−0.088	−0.028	0.108	−0.171^a^	−0.181^a^	0.147^a^	−0.057	0.063	0.092
SJ	0.022	0.035	0.230^a^	−0.066	−0.108	0.052	0.014	−0.089	0.067
SLJ	−0.522^a^	−0.159^a^	0.142	−0.559^a^	−0.041	0.201^a^	−0.587^a^	−0.184^a^	0.178^a^
PWC 170	0.298^a^	0.514^a^	0.213^a^	0.151^a^	0.480^a^	0.240^a^	0.128^a^	0.453^a^	0.136^a^
ST	0.058	0.059	0.097	0.038	−0.002	0.098	0.073	0.084	0.026
SES	−0.090	−0.062	0.020	−0.046	−0.113	−0.021	−0.083	−0.077	−0.022
*R* ^2^	29.3%	18.8%	13.7%	32.4%	28.6%	14.9%	31.4%	26.5%	30.8%

Abbreviations: FFMI, fat‐free mass index; FMI, fat mass index; MVPA, moderate to vigorous physical activity; PhA, phase angle; PWC170, physical working capacity 170; *R* ^2^, adjusted coefficient of determination; SES, socioeconomic status; SJ, side jump; SLJ, standing long jump; ST, screen time; yrs., years.

^a^ *ß* is significant at the 0.05 level.

**TABLE 3:** Unstandardized regression coefficients (*β*) of age, PA, PF, ST, and SES in relation to FMI, FFMI, and PhA among girls.

Girls	6–10 yrs.			11–13 yrs.			14–17 yrs.		
	FMI	FFMI	PhA	FMI	FFMI	PhA	FMI	FFMI	PhA
Model 0									
Age	0.264^a^	0.151^a^	0.040	0.237^a^	0.222^a^	0.149^a^	0.152^a^	0.149^a^	0.126^a^
*R* ^2^	6.7%	2.0%	0.0%	5.4%	4.7%	2.0%	2.2%	2.1%	1.4%
Model 0.1									
Age	0.580	0.576	1.811^a^	0.542	1.550	−2.806	1.196	−1.078	0.022
Age^2^	−0.317	−0.426	−1.776^a^	−0.306	−1.329	2.956	−1.045	1.227	0.104
*R* ^2^	6.5%	1.9%	1.4%	5.2%	4.7%	2.6%	2.1%	2.1%	1.3%
Model 1									
MVPA	−0.188^a^	−0.054	0.131^a^	−0.241^a^	−0.195^a^	−0.016	−0.079	0.095	0.107^a^
*R* ^2^	3.2%	0.0%	1.4%	5.5%	3.5%	0.0%	0.4%	0.7%	0.9%
Model 1.1									
Age	0.199^a^	0.095	0.050	0.194^a^	0.173^a^	0.123^a^	0.165^a^	0.172^a^	0.113^a^
MVPA	−0.144^a^	−0.033	0.143^a^	−0.200^a^	−0.158^a^	0.010	−0.059	0.117^a^	0.121^a^
*R* ^2^	6.7%	0.5%	1.3%	8.9%	6.1%	0.9%	2.8%	3.3%	1.9%
Model 2									
SJ	0.071	−0.096	0.042	−0.020	−0.020	−0.068	0.056	−0.038	−0.031
SLJ	−0.454^a^	−0.116	0.220^a^	−0.451^a^	−0.168^a^	0.163^a^	−0.472^a^	−0.119^a^	0.185^a^
PWC 170	0.356^a^	0.279^a^	0.003	0.273^a^	0.343^a^	0.004	0.186^a^	0.324^a^	0.096^a^
*R* ^2^	18.9%	5.8%	2.9%	24.2%	12.3%	1.2%	21.2%	11.0%	3.6%
Model 2.1									
Age	0.328^a^	−0.068	−0.227^a^	0.217^a^	0.160^a^	0.154^a^	0.106^a^	0.112^a^	0.141^a^
SJ	−0.043	−0.072	0.037	−0.045	−0.039	−0.086	0.035	−0.060	−0.058
SLJ	−0.521^a^	−0.102	0.266^a^	−0.445^a^	−0.163^a^	0.167^a^	−0.457^a^	−0.102^a^	0.206^a^
PWC 170	0.270^a^	0.297^a^	0.063	0.225^a^	0.308^a^	−0.029	0.171^a^	0.308^a^	0.077
*R* ^2^	24.9%	5.7%	5.5%	28.5%	14.6%	3.2%	22.1%	12.0%	5.3%
Model 3									
ST	0.250^a^	0.135^a^	−0.062	0.274^a^	0.172^a^	0.077	0.144^a^	0.081	−0.020
*R* ^2^	6.0%	1.6%	0.1%	7.3%	2.7%	0.4%	1.9%	0.5%	0.0%
Model 3.1									
Age	0.242^a^	0.151^a^	0.076	0.186^a^	0.211^a^	0.144^a^	0.154^a^	0.159^a^	0.132^a^
ST	0.203^a^	0.106^a^	−0.077	0.228^a^	0.120^a^	0.042	0.135^a^	0.072	−0.027
*R* ^2^	11.4%	3.5%	0.4%	10.4%	6.7%	2.1%	4.1%	2.8%	1.4%
Model 4									
SES	−0.197^a^	−0.165^a^	0.023	−0.216^a^	−0.183^a^	−0.031	−0.163^a^	−0.111^a^	−0.028
*R* ^2^	3.6%	2.5%	0.0%	4.5%	3.1%	0.0%	2.5%	1.1%	0.0%
Model 4.1									
Age	0.261^a^	0.145^a^	0.038	0.224^a^	0.211^a^	0.149^a^	0.149^a^	0.150^a^	0.128^a^
SES	−0.198^a^	−0.165^a^	0.023	−0.204^a^	−0.172^a^	−0.023	−0.157^a^	−0.104^a^	−0.022
*R* ^2^	10.2%	4.3%	0.0%	9.3%	7.4%	1.9%	4.6%	3.2%	1.4%
Model 5									
Age	0.311^a^	−0.089	−0.198	0.164^a^	0.123^a^	0.131^a^	0.114^a^	0.141^a^	0.145^a^
MVPA	−0.097	−0.049	0.066	−0.095	−0.135^a^	−0.032	−0.050	0.101	0.103
SJ	−0.039	−0.120	−0.037	−0.035	0.025	−0.090	0.006	−0.058	−0.044
SLJ	−0.487^a^	−0.025	0.282^a^	−0.360^a^	−0.080	0.131	−0.446^a^	−0.062	0.188^a^
PWC 170	0.202^a^	0.284^a^	0.075	0.183^a^	0.251^a^	−0.044	0.190^a^	0.258^a^	0.062
ST	0.059	0.036	−0.026	0.205^a^	0.091	−0.003	0.183^a^	0.127^a^	0.042
SES	−0.118	−0.138	−0.034	−0.069	−0.071	−0.018	−0.030	−0.097	−0.083
*R* ^2^	25.5%	5.6%	4.2%	29.1%	12.5%	0.8%	29.2%	14.2%	5.0%

Abbreviations: FFMI, fat‐free mass index; FMI, fat mass index; MVPA, moderate to vigorous physical activity; PhA, phase angle; PWC170, physical working capacity 170; *R* ^2^, adjusted coefficient of determination; SES, socioeconomic status; SJ, side jump; SLJ, standing long jump; ST, screen time; yrs., years.

^a^ *ß* is significant at the 0.05 level.

#### 3.3.1 6–10 years

In both sexes, FMI was positively associated with age, PWC170, and ST, but negatively with MVPA, standing long jump, and SES. For FFMI, positive associations were found with age, PWC170, and ST in both sexes, while boys showed a negative association with standing long jump and girls with SES. PhA showed positive associations with MVPA, standing long jump, and, for boys only, age and PWC170. Considering all predictors simultaneously in Model 5, associations with MVPA, SES, and ST lost significance for both sexes.

#### 3.3.2 11–13 years

FMI was negatively associated with age in boys but positively in girls. MVPA, standing long jump, and SES showed negative associations with FMI in both sexes. A positive association was found for PWC170 in both sexes, while for ST, a positive association was observed only in girls, with no association in boys. FFMI showed positive associations with age and PWC170, and negative associations with MVPA in both sexes. Additionally, in girls, there were negative associations with standing long jump and SES and a positive association with ST. PhA was positively associated with age in girls, with MVPA and PWC170 in boys, and with standing long jump in both sexes. In Model 5, MVPA remained significantly associated with FMI, FFMI, and PhA in boys, while in girls, only FFMI showed a significant negative association. The effects for SES and body composition parameters lost significance for both sexes.

#### 3.3.3 14–17 years

In both sexes, FMI was positively associated with age, PWC170, and ST, but negatively with standing long jump and SES. FFMI was positively associated with age and PWC170, but negatively with standing long jump and SES in both sexes, while MVPA showed a positive association with FFMI only in boys. PhA was positively associated with age, MVPA, standing long jump, and PWC170 in both sexes. In Model 5, all effects for MVPA, ST, and SES lost significance in boys, while in girls, only MVPA and SES lost significance.

Table 4 provides a summary of the confirmed and rejected hypotheses based on the significance levels presented in Tables 2 and 3.

**TABLE 4:** Confirmation or rejection of the hypotheses.

Hypotheses	6–10 yrs.		11–13 yrs.		14–17 yrs.	
	Boys	Girls	Boys	Girls	Boys	Girls
H1 (MVPA, FMI, r−^a^)	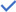 ^b^	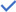	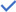	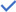	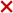 ^c^	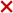
H2 (MVPA, FFMI, r+^d^)	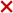	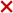	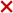	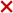	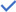	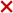
H3 (MVPA, PhA, r+)	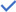	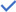	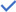	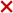	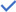	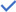
H4 (PWC170, FMI, r−)	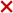	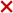	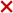	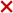	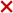	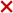
H5 (PWC170, FFMI, r+)	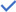	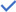	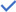	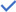	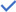	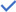
H6 (PWC170, PhA, r+)	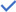	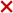	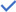	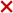	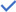	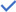
H7 (SLJ, FMI, r−)	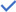	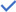	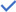	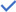	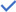	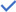
H8 (SLJ, FFMI, r+)	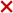	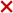	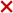	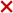	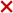	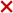
H9 (SLJ, PhA, r+)	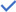	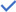	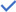	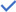	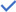	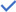
H10 (SJ, FMI, r−)	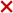	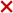	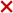	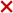	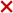	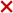
H11 (SJ, FFMI, r+)	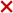	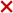	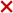	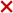	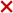	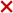
H12 (SJ, PhA, r+)	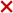	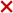	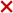	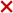	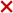	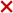
H13 (ST, FMI, r+)	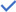	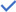	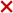	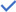	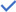	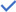
H14 (ST, FFMI, r−)	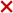	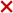	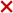	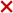	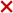	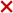
H15 (ST, PhA, r−)	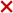	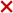	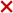	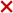	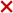	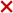
H16 (SES, FMI, r−)	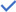	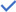	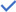	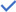	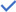	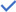
H17 (SES, FFMI, r+)	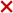	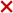	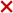	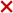	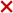	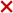
H18 (SES, PhA, r+)	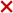	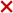	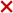	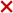	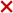	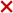

Abbreviations: FFMI, fat‐free mass index; FMI, fat mass index; H1‐H18, hypotheses formulated prior to the study; MVPA, moderate to vigorous physical activity; PhA, phase angle; PWC170, physical working capacity 170; *r*, measure of association; SES, socioeconomic status; SJ, side jump; SLJ, standing long jump; ST, screen time.

^a^ Negative directed hypothesis.

^b^ Hypothesis confirmed in isolated approach.

^c^ Hypothesis rejected in isolated approach.

^d^ Positive directed hypothesis.

In total, 43 hypotheses were confirmed and 65 were rejected based on the models 1–4. Contrary to expectations, we observed positive associations between PWC170 and FMI in both sexes across all age groups. Regarding the standing long jump, we found either no association or negative associations with FFMI in both sexes across all age groups. Additionally, the side jump was not associated with FMI, FFMI, or PhA in our sample. Lastly, the predicted negative association between ST and FFMI and PhA could not be confirmed in our study, nor could the positive association between SES and FFMI and PhA.

## 4 Discussion

Our study's objective was to depict and analyze associations of body composition during childhood and adolescence using a cross‐sectional nationwide sample from a German longitudinal study. The data show, that both sexes increase their FMI, FFMI, and PhA with age. Boys show an accelerated increase in FFMI starting approximately at the age of 11 and an accelerated increase in PhA starting around 14 years. FMI increases at a similar rate in both sexes until approximately the age of 10. Starting at the age of 11, girls show an increase in FMI compared to boys, who exhibit a decline in FMI between the ages of 11 and 13.

Alongside age, PWC170 and standing long jump were the strongest predictors of FMI and FFMI in both sexes, as well as PhA among boys. Regarding PA, associations remained significant in the multivariate models only among the 11 to 13‐year‐old boys for FMI, FFMI, and PhA. For girls, significant associations were observed only for FFMI. This timeframe may represent a sensitive period for the effects of PA to manifest on body composition, particularly among boys, during childhood. For ST, girls showed significant positive associations with FMI across all age groups and significant positive associations with FFMI between the ages of 6 and 13 years. Among boys, we found only three significant associations with FMI and FFMI between the ages of 6 and 13, of which none remained significant in the multivariate models. There were no significant associations for SES in the multivariate models. These findings highlight the importance of considering multiple theory‐based predictors to limit the problem of overstated or confounded results.

### 4.1 FMI

Due to hormonal adaptations, childhood and adolescence involve the most sensitive phases in development, characterized by growth and maturation, leading to inevitable changes in body composition (Marshal, 1978; Rogol 2003). We found positive associations between age and FMI for all age groups except for boys aged between 11 and 13 years where a negative relationship between age and FMI was found. In a study of Siervogel et al. (2000) with 297 children and adolescents between the ages of 8 and 18, FMI increased between the ages of 9 and 11 for both sexes, then only boys showed a decrease in FMI between the ages of 12 and 17. In boys, the influence of sexual hormones leads to a significant increase in bone and muscle mass, as well as a redistribution (Rogol 2003) and reduction (Marshall 1978) of fat mass. However, as height velocity declines, fat accumulation resumes (Marshall 1978).

Besides genetically triggered age effects, PA is known to be crucial for maintaining a healthy body composition during all stages of life (Gualdi‐Russo et al. 2020; Westerterp 2018). The results of our study confirm our hypotheses that higher MVPA is associated with lower FMI at ages 6–13 in both sexes. However, this association becomes weaker at ages between 14 and 17 years for both sexes, where our hypotheses were not fulfilled. A possible reason for this is the fact that PA generally decreases at the end of puberty, not only in humans, but nearly all mammals (A. E. Chung et al. 2012; Westerterp 2013). Another explanation might be that other parameters of lifestyle, such as individual diet and work‐related activity take the determine role in defining body composition after puberty and graduating from school.

Concerning ST, except for boys aged 11–13 years, positive associations between ST and FMI were found for all age groups, suggesting that our hypotheses were generally supported. A recent meta‐analysis among children also showed a positive association between total ST and overweight/obesity, with an increased risk in children who spent more than 2 h per day on screen‐based devices (Fang et al. 2019). Another recent review also showed positive associations for body fat percentage with ST in children and adolescents (Tripathi and Mishra 2020). This may be triggered by unconscious eating behavior while watching television (Borgogna et al. 2015) or using a smartphone (La Marra et al. 2020), and the consumption of high‐calorie snacks, sometimes triggered by commercials, during ST (Borgogna et al. 2015; La Marra et al. 2020). However, studies also show that there is no general negative effect of ST on FMI and that it rather depends on the type of ST and the individual (Christofaro et al. 2016; Falbe et al. 2013; Marker et al. 2019).

PF also benefits from an active lifestyle (Mateo‐Orcajada et al. 2022) and is associated with body composition in childhood and adolescence (Ortega et al. 2008; Smith et al. 2014). Our study confirms this but also shows that the extent and direction of the association depends on how PF is operationalized and measured. For example, the standing long jump performance shows negative associations with FMI for both sexes and all age groups, thereby confirming our hypotheses comprehensively. Standing long jump is a functional test to measure the explosive power of the lower extremities (Mackala et al. 2013). Here, individual performance depends not only on raw strength, but also on coordination (Ashby and Delp 2006), jumping technique (Mackala et al. 2013), and body weight (Henriksson et al. 2022). A longitudinal study of 240 adolescent girls showed that fat mass was inversely associated with standing long jump (Kasović et al. 2022). Another study also showed negative associations for FMI with standing long jump in 9‐year‐old children (Henriksson et al. 2022). A closer look at the study's findings reveals that only lower extremity strength (standing long jump, weight‐bearing) is negatively associated with FMI, while a positive association was measured for upper extremity strength (hand grip, non‐weight‐bearing). Another cross‐sectional study of 6 to 18‐year‐olds showed that higher handgrip strength (non‐weight‐bearing) is found in those who are generally taller and weigh more (Dos Santos de Fontes et al. 2023). These findings may also explain why positive associations between FMI and PWC170 measured by a non‐weight‐bearing ergometer test were found in the present study, contrary to our initial hypotheses. Leg muscle mass and its energy supply are key factors for the PWC170 cycling ergometer test, and both benefit from higher overall body weight and the inevitable daily work capacity of the lower extremities that comes along with it. For the present study, we also calculated regression analyses using PWC170 standardized for body weight, which then showed expected negative associations for both sexes and all age groups. [6–10 years boys: *β* = −0.336, girls: *β* = −0.455; 11–13 years boys: *β* = −0.358, girls: *β* = −0.401; 14–17 years boys: *β* = −0.320, girls: *β* = −0.280]. In daily life, higher PF is often associated with lower fat mass and higher muscle mass, but it is important to consider that non‐weight‐bearing performance can be positively correlated with fat mass. Regarding coordination under time pressure, our study did not reveal significant relationships with FMI for either sex, which contradicts our initial hypotheses.

In addition to the aforementioned factors associated with body composition, our study showed negative associations between FMI and SES for all age groups and both sexes, aligning with our hypothesized relationships. This is consistent with the findings of a recent review showing negative associations between fat mass and SES in children living in high‐income countries (Bridger Staatz et al. 2021; Vazquez and Cubbin 2020). In this context, PA and eating patterns were discussed as key predictors of the results. This is supported by another review's findings revealing that children and adolescents from higher SES backgrounds are more active and consume healthier food than children and adolescents from lower SES backgrounds (Gautam et al. 2023).

Considering the results from the multivariate models, it becomes evident that the effects of age, PWC170, and standing long jump consistently remained significant, while the effects of MVPA, SES, and ST mostly lost significance. The *R* ^2^ of the respective models underline this finding, as they clearly show that the fitness variables and age explain the largest part of the variance.

### 4.2 FFMI and PhA

BIA derived FFMI is used as a predictor for the quantity of active cell mass and when compared to individuals with similar age, sex, and ethnicity, a predictor of the quantity of muscle mass (Kawakami et al. 2022). PhA is directly derived from impedance and resistance and is associated with cellularity, cell hydration, and integrity of the cell membrane (Bosy‐Westphal et al. 2006). Therefore, it can be used as a predictor of both, quality (e.g., membrane permeability and nutritional status) and quantity of muscle mass (Martins et al. 2023).

In children and adolescents, sex, age, and body mass index are considered the main determinants of FFMI and PhA (Bosy‐Westphal et al. 2006; Gualdi‐Russo et al. 2020; Kasović et al. 2021). According to a review carried out by Gualdi‐Russo et al. (2020), in addition to being male and increasing age, less ST was also positively associated with FFMI. MVPA showed no association with FFMI in their analysis (Gualdi‐Russo et al. 2020). We also found no clear association between FFMI and MVPA in our study, which did not support our hypothesized relationship. In an intervention study carried out among Estonian children aged 6 to 8, the authors found that substituting 5 minutes per day of sedentary behavior at 6.6 years with 5 minutes of vigorous PA was related to higher FFMI at the age of 7.6 (Reisberg et al. 2020). In general, the literature suggests that the relationship between parameters of body composition and PA is inconclusive between PA intensity levels with the strongest relationships found for vigorous PA (Gralla et al. 2019). In a review by Mundstock et al. (2019), the authors found that in cross‐sectional studies, the PhA was on average 0.70° higher among active individuals (95% CI: 0.48–0.92), compared to less active or inactive individuals. The authors also considered the baseline PhA of selected longitudinal studies and found a mean difference of 0.30° between the active groups and the control groups (95% CI: 0.11–0.49) (Mundstock et al. 2019). We also found a significant positive association between PhA and MVPA among boys of all age groups and girls aged 6–10 and 14–17, aligning with our hypotheses for these groups. Whereas FMI and FFMI are simple parameters of quantity, PhA also depends on cellular integrity (Langer et al. 2020; Stobäus et al. 2010), which can be positively influenced by MVPA. This confirms the PhA as an important biomarker besides FMI and FFMI (Stobäus et al. 2010).

In a recent study carried out among 12.678 adolescents aged 11 to 18 in Croatia, the authors found that FFMI generally increases with age in boys while girls show more stable FFMI across adolescents (Kasović et al. 2021). Besides the fact that boys are more physically active during adolescence, for example in sports clubs and regarding playing outside (Schmidt et al. 2020b), these differences are for the most part due to hormonal changes during puberty (Loomba‐Albrecht and Styne 2009; Siervogel et al. 2003).

Contrary to our hypotheses, we found no significant relationships between FFMI or PhA and side jump among both sexes. During the side jump, the body's center of gravity changes only slightly in vertical position, theoretically placing it between a weight‐bearing and a non‐weight‐bearing motor performance task. Future research is needed to further investigate the interplay between body composition and coordination tasks. Nevertheless, we observed positive associations between both, FFMI and PhA, with PWC170 among boys, confirming the related hypotheses. These findings are consistent with a study by Langer et al. (2020), where significant correlations between PhA, fat‐free mass, and submaximal oxygen uptake are reported. Among girls, only FFMI exhibited a significant positive association with PWC170 across all age groups, thereby confirming our hypotheses. In contrast, PhA showed a significant association with PWC170 solely in the 14–17 age group, thereby partially supporting our related hypotheses.

Standing long jump showed a significant positive association with PhA among boys and girls of all age groups, whereas FFMI was negatively associated with it. Other studies also found similar significant relationships between FFMI and PF for children and adolescents (Avcin et al. 2023; Henriksson et al. 2016). In contrast to the PWC170, the standing long jump is a weight‐bearing task. FFMI includes muscle mass across the entire body, including the upper extremities, which contribute less to performance in a weight‐bearing activity like the standing long jump. Additionally, higher fat mass may accompany greater muscle mass, but this does not necessarily translate to better performance in weight‐bearing activities such as the standing long jump. These factors may explain why FFMI was not positively associated with standing long jump performance in our study.

Considering the results from the multivariate models, similar to FMI, the effects of age, PWC170, and standing long jump remained significant while the effects of MVPA, SES, and ST largely diminished or varied. This is further supported by the amount of explained variance in the models, especially for FFMI.

Our study's strengths include the use of a nationwide sample. Additionally, we employed high‐standardized methods, such as BIA and accelerometry to measure body composition and PA. A comprehensive motor performance test profile was utilized to assess specific PF parameters. Our study also has limitations. Despite the comprehensive inclusion of multiple variables, the consideration of nutrition was notably absent. This omission holds significance as nutrition plays a pivotal role in the growth and development of children and adolescents (Corkins et al. 2016), thereby potentially exerting an influence on our study findings. We also did not include maturation status to stratify our sample. Standard indicators of maturation include hormonal changes, breast tissue development, and testicular enlargement (Cheuiche et al. 2021), none of which were measured in the MoMo study. Causal relationships cannot be inferred from the results and the found associations should be interpreted with caution due to the cross‐sectional design. We used a broad time span throughout the day to test our subjects. There is evidence that motor performance varies slightly throughout the day and correlates with circadian rhythm (Atkinson and Reilly 1996; Edwards et al. 2007). However, these variations are small, more pronounced in trained persons and athletes and literature suggests that they should be recognized especially when focusing on repeated‐measurement designs. Therefore, we did not make any adjustments for circadian rhythm in our study. The MoMo study does not collect data on race or ethnicity. This clearly limits the comparability of our findings with international studies and affects the generalizability to children and adolescents outside of Germany. Our study is designed to be representative of children and adolescents living in Germany, which defines and delimits the scope of our results. This should be considered when interpreting the findings. Finally, it's crucial to acknowledge that how fitness variables are expressed, whether in absolute or relative terms, dictates the direction of their association with parameters of body composition. This aspect complicates the comparison with findings from other studies.

## 5 Conclusion

Our results highlight the complex interplay between body composition, PF parameters, and lifestyle factors in childhood and adolescence. Variations in findings across studies may be due to differences in how PF and other parameters are defined, for example, whether PF is assessed using non‐weight‐bearing or weight‐bearing tasks. Furthermore, we demonstrate that data from BIA can be used to describe developmental changes in body composition during childhood and provide an overview of its associations with other health‐relevant parameters in a nationwide German sample. During childhood, both sexes increase their FMI, FFMI, and PhA. At the age of approximately 11, boys show an accelerated increase in FFMI and a slightly time‐shifted accelerated increase in PhA. This is paralleled by a decline in FMI among boys whereas girls increase their FMI, FFMI, and PhA more or less linear.

Concerning body composition correlates, the conducted regression analyses showed that primarily PWC170 and standing long jump performance are related to FMI and FFMI in both sexes, as well as PhA in boys.

Body composition growth charts carried out by BIA could be a solution to the need for a non‐invasive and economical assessment and monitoring of body composition. Monitoring body composition through the entire life course with a feasible method such as BIA could ultimately help scientists to better understand the association between growth, aging, health, and disease (S. Chung 2019). It could also help physicians, for example during preventive check‐ups or for assessing biological age, and ultimately individuals to track and maintain a healthy body composition against the multitude of external and internal influencing factors throughout the lifespan.

## Ethics Statement

On September 23, 2014, the ethics committee of the Karlsruhe Institute of Technology approved the study.

## Consent

Written informed consent to participate in this study was provided by the participants' legal guardian/next of kin.

## Conflicts of Interest

The authors declare no conflicts of interest.

## Data Availability

Data cannot be shared publicly because of strict ethical conditions with which study investigators are obliged to comply: The Charite/Universitätsmedizin Berlin ethics committee and the Federal Office for the Protection of Data explicitly forbid making the data publicly available because informed consent from study participants did not cover public deposition of data. However, the minimal data set underlying the findings is archived at the Institute of Sports and Sports Science of the Karlsruhe Institute of Technology (KIT) and can be accessed by interested researchers on‐site. Access requests should be submitted to the Institute of Sports and Sports Science, Karlsruhe Institute of Technology, Engler‐Bunte‐Ring 15, 76131 Karlsruhe, Germany.
